# Suspected Palytoxin Inhalation Exposures Associated with Zoanthid Corals in Aquarium Shops and Homes — Alaska, 2012–2014

**DOI:** 10.15585/mmwr.mm6431a4

**Published:** 2015-08-14

**Authors:** Ali K. Hamade, Sandrine E. Deglin, Joe B. McLaughlin, Jonathan R. Deeds, Sara M. Handy, Ann M. Knolhoff

**Affiliations:** 1 Section of Epidemiology, Division of Public Health, State of Alaska; 2 Center for Food Safety and Applied Nutrition, U.S. Food and Drug Administration

On August 12, 2014, an Anchorage hospital notified the Alaska Section of Epidemiology (SOE) that a middle-aged male resident of Anchorage (patient A) had arrived in the emergency department with possible palytoxin exposure. Patient A complained of a bitter metallic taste, fever, weakness, cough, and muscle pain 7–8 hours after introduction of live zoanthid coral into his home aquarium. Palytoxin, a potent toxin known to produce the reported effects, is contained in zoanthid marine corals (1,2).

This call prompted SOE to launch an epidemiologic investigation, during which investigators interviewed exposed persons, obtained environmental specimens for testing, and provided advice about avoiding continued exposure. Patient A reported that two persons (patients B and C) who lived with him experienced similar symptoms around the same time. Patient A also reported that the owner of a local aquarium shop knew of numerous reported aquarium-related poisonings associated with suspected palytoxin-containing zoanthids, both through personal experience and through online marine aquarium forums (3). Patient A reported that the shop’s owner believed that he and several of his employees and customers had been previously exposed, some multiple times.

A specimen obtained from patient A’s introduced coral, as well as a specimen obtained from the shop, were both positive for palytoxin. An extended investigation identified seven additional Anchorage residents who appeared to have experienced acute palytoxin-related illness during the preceding 2 years. Many aquarium store employees and marine aquarium hobbyists are not aware of palytoxin as a potentially serious hazard associated with handling some zoanthid corals sold in aquarium stores or exchanged by hobbyists. Persons who are likely to handle such organisms should be made aware of the potential health risks so that they understand how to prevent exposure to this potent toxin.

## Case Reports

On August 11, 2014, at 10:30 p.m., a relative of patient A transferred 70 pounds (32 kg) of live coral from a plastic container into patient A’s 200-gallon (758-L) aquarium in his 1,600-square-foot (149 square-meter) mobile home. During the transfer, several coral fragments fell to the floor, causing some of the live polyps to break off. Patients B and C were asleep in an adjacent room <20 feet [<6 m] from the aquarium while the coral was being transferred. Patient A arrived home at 11:30 p.m. and slept for approximately 7 hours in the room with the aquarium. On August 12, at approximately 7:00 a.m., patients A, B, and C awoke with neurologic, respiratory, musculoskeletal, and other symptoms (Table). Because of the severity of patient A’s symptoms, which included cough, nausea, headache, and muscle and joint pain, he was taken to a nearby hospital emergency department, where he was tachycardic, tachypneic, and febrile (maximum temperature = 103°F [39.4°C]). His white blood cell count was elevated at 13,800 cells/cubic milliliter with 86% neutrophils. His renal function tests, urinalysis, troponin I, creatinine kinase, and chest radiograph were unremarkable. Influenza A and B tests were negative. He was admitted to the hospital for supportive care. Patients B and C gradually improved throughout the day and their symptoms completely resolved by 7:00 p.m. Patient A was released 2 days later, after resolution of his symptoms. The person who introduced the coral into the aquarium was reported to be asymptomatic.

Patient A stated that the household dog had vomited the morning after coral introduction (August 12) and both the dog and the household cat appeared to be lethargic that day. Patients A and C noted a visible mist and sensed humidity in the mobile home on the morning after coral introduction, leading them to suspect a possible problem with the aquarium. The patients reported learning that palytoxin was a possible cause of their illness from the owner of the shop. The shop owner stated that he had experienced similar symptoms on multiple occasions after handling zoanthid corals, and that he had read numerous similar reports posted by other marine aquarium enthusiasts through online blogs (3). SOE advised patients A, B, and C to decontaminate surfaces near the aquarium with dilute household bleach while wearing personal protective equipment including face mask, goggles, and overalls.

## Laboratory Analysis

SOE arranged with the U.S. Food and Drug Administration Center for Food Safety and Applied Nutrition to test coral samples from the shop and from the aquarium in patient A’s house. Three samples from the shop and two samples from the home of patient A were selected on the basis of visual resemblance to zoanthids previously reported to contain palytoxin (2). Quantitative analysis was performed using high performance liquid chromatography with ultraviolet detection compared against a palytoxin standard (2). The analysis confirmed 7.3 mg crude palytoxin/g wet weight of zoanthid tissue in one coral sample from patient A’s home aquarium (Figure) and 6.2 mg crude palytoxin/g wet weight zoanthid in one coral sample from the shop. The three additional coral samples were nontoxic or only weakly toxic. The levels of palytoxin in the corals exceeded those found in investigations of previous similar poisoning events (0.5 mg/g–3.5 mg/g) (2). An additional analysis by high resolution liquid chromatography mass spectrometry (2) confirmed that the primary toxin in both samples was palytoxin (molecular weight = 2,680 kilodaltons). Genetic analysis (2) determined that both toxin-containing zoanthid samples were consistent with previous molecular identifications of a highly toxic variety of *Palythoa* species collected from multiple aquarium shops in Maryland and Virginia, and from three similar aquarium-related poisoning events in New York, Ohio, and Virginia. Both specimens were genetically and visually distinct from the nontoxic or weakly toxic specimens from this case and similar previous cases.

## Additional Case Reports

SOE followed up with the owner of the shop to identify additional cases. He reported that he and several aquarium shop staff members had experienced numerous episodes of likely palytoxin poisoning resulting in acute onset of clinically compatible symptoms (Table). The most recent recalled incident occurred in July 2014, and involved seven staff members who were exposed either while dismantling a customer’s private aquarium containing corals or upon later handling of the aquarium contents at the shop. SOE interviewed four of the staff and the shop owner (patients D, E, F, G, and H). All reported experiencing a bitter metallic or salty taste within 2 hours of exposure, followed by one or more of the following: cough, joint pain, flank pain, fever, and cold sensation during the night. Signs and symptoms largely resolved by the following morning (Table). Possible palytoxin exposure occurred while mouth-siphoning water out of the aquarium, and transporting and handling coral rocks that were exposed to air. Two staff members reported experiencing similar symptoms several weeks after the July 2014 event, after handling the same corals out of water and after cleaning dry plastic pipes from the aquarium with hot water.

Several staff members reported symptoms consistent with palytoxin exposure on multiple occasions; one had experienced such symptoms nine times. SOE was able to interview only five shop staff members; however, at least three others were reportedly exposed to palytoxin. Subjects reported managing their symptoms by increasing intake of fluids. SOE provided information to shop staff on how to detoxify palytoxin on surfaces using diluted household bleach.

The owner of the shop notified SOE of two additional suspected palytoxin poisonings in an Anchorage household in 2012. These two persons (patients I and J) reported fever, tremors, weakness, ataxia, and other symptoms (Table) within hours of cleaning a fish tank that contained zoanthids. Both patients were hospitalized in the intensive care unit for several days. Patient I, who was pregnant at the time, experienced preterm labor the day after her hospital admission and delivered her baby at 6 months’ gestational age. The child survived and reportedly suffered no apparent long-term adverse health effects. Patient J reported lingering pulmonary effects 2 years after exposure. Palytoxin exposure likely occurred after patient J cut polyps away from their rock base under hot water in the home garage; his wife (patient I) and dog walked through the garage several times during the process. The dog reportedly vomited and was lethargic following the tank cleaning.

**Summary**

What is already known on this topic?

Palytoxin is a potentially life-threatening toxin that can act via dermal, inhalation, and oral routes of exposure. Marine aquarium hobbyists who introduce certain zoanthid corals into their aquariums are at risk for palytoxin exposure.

What is added by this report?

At least ten persons in Alaska developed signs and symptoms compatible with palytoxin exposure after either handling zoanthid corals or being in proximity to someone who did.

What are the implications for public health practice?

The risks for palytoxin exposure are unknown to many in the commercial aquarium and hobbyist communities. Activities that could potentially produce aerosols (e.g., scrubbing or using hot water to remove zoanthids) should be undertaken with caution. Hobbyist and commercial coral growers and the public health and health care provider communities might benefit from common recommendations on coral handling and decontamination practices from state and federal public health agencies. Illnesses after a potential exposure should be promptly reported to the state or local health department.

### Discussion

Palytoxin is a potent vasoconstrictor that acts by binding to Na+/K+ ATPase, which leads to destruction of the ion gradient across cell membranes, passive transport of ions, and ultimately, cell death (4). It causes a range of effects in animals and humans, depending on the route of exposure (5,6). The dose at which 50% of exposed animals die following intravenous administration of palytoxin (LD50) has been shown to be as low as 0.033 *μ*g/kg body weight (6). Higher concentrations are required to cause effects following incidental contact depending on whether the exposure occurs through dermal, inhalation, or oral routes (5). Based on reports in the medical literature (7) and online forums (3), most aquarium-related exposures occur after subjecting zoanthids to prolonged handling and appear to be related to inhalation or to skin exposures through cuts on the hands and fingers in persons who maintain these types of aquariums. Throughout the Mediterranean region, palytoxin exposure has been linked to fever, conjunctivitis, and respiratory symptoms in persons exposed to marine aerosols during proliferations of palytoxin and palytoxin-like compound–producing marine algae (i.e., algal blooms) (5), but detailed inhalation studies in animal models are lacking. No antidote is available for palytoxin; treatment is supportive.

Zoanthids (Class Anthozoa, Subclass Hexacorallia, Order Zoanthidia [colonial anemones]) are common in home aquariums. They are considered relatively easy to keep and are often recommended to new aquarium owners. Some types of colonial anemones form large aggregations encrusting a hard substrate. In an aquarium, these aggregations often require thinning or removal. Because of the way these organisms attach to surfaces, aggressive methods are sometimes required for their removal, including cutting, scraping, applying chemicals, or scalding with hot water, which lead to an increased potential for palytoxin exposure, often through the presumed production of aerosols (7). Other potential exposure routes include direct contact with eyes, through skin lesions, and incidental ingestion. Although not all zoanthids contain palytoxin, some zoanthids commonly found in home aquariums contain high concentrations of this toxin (2). Some coral enthusiasts appear to be able to maintain them without ill effects, likely through proper handling, aquarium management, and decontamination practices. Palytoxin can be neutralized by soaking the coral for 30 minutes in a ≥0.1% household bleach solution (1 part 5%–6% sodium hypochlorite [household bleach] to 10 parts water, prepared fresh) (8). Contaminated items should be soaked in diluted bleach before disposal (3).

Palytoxin is known to some coral hobbyists (3), and the Anchorage aquarium shop displayed many signs warning that some coral might be very toxic. However, no U.S. regulations govern the testing or labeling of coral that might contain toxins, including palytoxin. Regulations for the importation of corals currently enforced by the U.S. Fish and Wildlife Service pertain to endangered species and reflect ecological concerns (9). General recommendations on coral handling and decontamination practices would be helpful for hobbyists, commercial coral growers, and the public health and clinical provider communities.

Currently, no official evidence-based recommendations exist for proper personal protective equipment use for coral hobbyists and aquarium shop staff, and development of such recommendations might be helpful. Activities that could potentially produce aerosols (e.g., scrubbing or using hot water to remove zoanthids) should be undertaken with caution. Patients A, B, and C did not handle any of the corals directly; rather, they were present in the home shortly after the introduction of palytoxin-containing zoanthids to the aquarium. Until data from controlled inhalation experiments in an animal model are available, this apparent link between palytoxin and inhalation toxicity will remain associative and evidence-based recommendations on appropriate respiratory protection or handling best practices will not be possible.

## Figures and Tables

**FIGURE:**
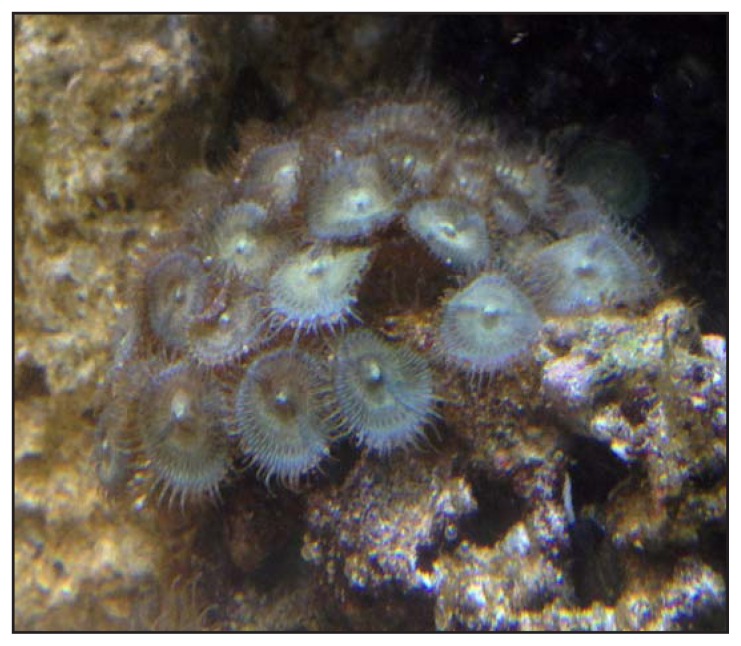
Zoanthid colony associated with palytoxin toxicity in patients A, B, and C, collected from a home aquarium — Anchorage, Alaska, August 2014

**TABLE:** Characteristics of patients in reported and investigated cases of palytoxin poisonings — Anchorage, Alaska, 2012–2014

	Patient									
	
Characteristic	A	B	C	D	E	F	G	H	I	J
**Outbreak no.**	1	1	1	2	2	2	2	2	3	3
**Patient sex, age (yrs)**	M, 32	F, 30	M, 50	M, 43	M, 24	M, 52	M, 24	M, 40s	F, 29	M, 32
**Year exposed**	2014	2014	2014	2014	2014	2014	2014	2014	2012	2012
**Symptoms**										
Bitter metallic taste	x		x	x	x	x	x		x	x
Salty taste		x			x		x			
Paresthesia	x	x	x		x				x	
Nausea	x	x	x					x	x	
Vomiting									x	
Weakness	x	x	x	x	x			x	x	x
Ataxia	x		x						x	x
Muscle spasms	x		x						x	
Loss of appetite	x		x							
Dyspnea	x	x	x	x			x	Unsure	x	x
Headache		x							x	
Cough		x	x	x			x			
Scratchy throat	x	x	x						x	x
Joint/muscle pain	x	x	x	x			x	x	x	
Fever	x	x	x	x	x		x	x	x	x
Tremors	x	x	x	x	x	x	x	x	x	x
Dry mouth/throat		x	x							
Kidney pain	x		x	x	x					
Dysphagia			x						x	
Dizziness			x						x	x
**Times exposed**	1	1	1	6–8	1	2–3	1	9	1	1
**Additional reported symptoms/signs**	Lungs “on fire”; light sensitivity; tachycardia (135 bpm); fever (103°F); BP 118/69; 96% O_2_ saturation	Lungs “heavy, compressed”; raspy voice; painful swallowing	Nose bleed; floating sensation		“Pulmonary congestion”				Inhaler used for 4 weeks after 5-day hospitalization (3 days in ICU)	Dysphonia; dysarthria; hyperventilation; anoxia (low [34%] O_2_ saturation); loss of consciousness; inhaler use for 4 weeks after 9-day hospitalization (5 days in ICU); full recovery of aerobic capacity incomplete 2 years after exposure (self-reported)

**Abbreviation:** ICU = intensive care unit, O_2_ = oxygen.
